# Comment on Othman et al. Ventricular Topology in Congenital Heart Defects Associated with Heterotaxy: Can We Find Patterns Reflecting the Syndrome-Specific Tendency for Visceral Symmetry? *J. Cardiovasc. Dev. Dis.* 2025, *12*, 430

**DOI:** 10.3390/jcdd13020067

**Published:** 2026-01-27

**Authors:** Flaminia Pugnaloni, Giulio Calcagni, Bruno Marino

**Affiliations:** 1 Department of Maternal Infantile and Urological Sciences, Sapienza University of Rome, 00185 Rome, Italy; bruno.marino@uniroma1.it; 2 Neonatal Intensive Care Unit, “Bambino Gesù” Children’s Hospital IRCCS, 00165 Rome, Italy; 3 Clinical Cardiology Unit, Division of Cardiac Surgery, “Bambino Gesù” Children’s Hospital IRCCS, 00165 Rome, Italy; giulio.calcagni@opbg.net

We read with great interest the recent and important paper by Othman et al. [1], which reports significant correlations with cardiovascular phenotypes in cases with heterotaxy (HTx). An anatomic, embryologic, and genetic approach, such as the one employed by the authors, is essential for the study of complex congenital heart defects (CHDs), particularly in cases of HTx. The anatomic correlation identified are clinically useful to better understand this complex topic.

The authors of the present paper [1] correctly confirms the prevalence of transposition of great arteries (TGA) in patients with Htx and right isomerism of the atrial appendages (RI) compared to cases with left isomerism of the atrial appendages (LI) [2].

However, we would like to comment that, in our opinion, the use of the generic term “double outlet right ventricle” (DORV) to describe the outflow tract of these patients is insufficient and potentially misleading. DORV, accordingly with the sequential segmental analysis [3], defines a ventriculo-arterial connection but, by itself, does not represent a precise anatomic description of the cardiac outflow tracts. The strict application of segmental analysis may be considered a practical approach to diagnose CHD but is not adequate and may be confounding for detailed anatomic and morphogenetic investigation of cardiac defects.

In fact, the spectrum of DORV encompasses both cases with aortic dextroposition with posterior aorta, subpulmonary infundibulum, and normally spiralized and related great arteries and cases with an anterior and right or left-sided aorta associated with a subaortic or bilateral infundibulum.

These two types of cardiac defects are clearly distinct from both anatomical and embryogenetic perspectives [4,5]. For morphogenetic studies, an accurate description of infundibular morphology is more informative than sequential analysis based exclusively on the classification of ventriculo-arterial connections.

It is well known that in patients with Htx with RI in addition to TGA, many cases of DORV also occur, usually characterized by an anterior aorta surrounded by a muscular infundibulum with parallel and non-spiralized great arteries [6]. Moreover, also in patients with RI and pulmonary atresia (“single outlet”), the aorta is constantly anterior with a muscular infundibulum.

Therefore, in the great majority of cases with RI, the outflow tract morphology corresponds to TGA or, alternatively, to DORV or single outlet, which, despite sequential segmental analysis, exhibit anatomical and morphogenetic characteristics similar to those of TGA.

*Pitx2c* is a major lateralization gene and in *Pitx2δc* mutant embryos it has been showed a disruption of the normal rotational migration of the pulmonary infundibulum, causing TGA or DORV with parallel and non-spiralized great arteries [7]. This represents one of the strong primary links between RI and some cases of TGA. The grow deficit of the arterial pole [8] may be considered an interesting additional or alternative mechanism causing, together with other morphogenetic defects, TGA.

On the contrary, as clearly described by Othman et al. [1] and widely reported in the literature [9,10] in the majority of cases with Htx and left atrial appendages isomerism (LI), the great arteries are normally connected to the ventricles and normally related to each other, exhibiting clockwise spiraling (seen from the apex) in cases with a D-ventricular loop. But also in cases with LI and L-ventricular loop, the great arteries are frequently normally connected and inversely normally related (mirror image) with counterclockwise spiraling (seen from the apex) [2,9,10,11]. The frequent ventriculo-arterial concordance (in both D- and L-loop) may explain, together with more balanced ventricular anatomy, the better surgical prognosis of LI [1,11].

Thus, in LI, cardiac ambiguity is frequently limited to the atria and the venous connections, whereas the ventriculo-arterial patterns are similar to those observed in situs solitus or situs inversus [2]. This intriguing aspect, in our opinion, warrants further investigation to better understand the differences between RI and LI.

Segmental cardiac disharmony in cases of RI and LI is not surprising and resembles other forms of disharmony characterized by discordance between cardiac segments in patients with situs solitus or situs inversus. For example, TGA represents a disharmony between normal (solitus) atrial situs with normal D-ventricular loop but with discordant ventriculo-arterial connection. Similarly, congenitally corrected TGA reflects disharmony between atrial situs solitus and discordant L ventricular loop with discordant ventriculo-arterial connection. For these disharmonic and discordant cardiac phenotypes, the involvement of ciliary and/or lateralization genes has been suggested in some cases [12] and has also been demonstrated [13,14]. Lateralization genes typically exert homogeneous effects on the organism; however, they may occasionally produce different, organ-specific effects [15] and can also independently influence distinct cardiac segments [16].

In conclusion, DORV, in accordance with sequential segmental analysis [3], defines a ventriculo-arterial connection but, by itself, does not provide a precise anatomical description of the cardiac outflow tracts. The fascinating complexity of Htx-related cardiac defects does not involve only atrial appendage anatomy [9] and the ventricular loop [1]; rather, the anatomy of the outflow tract in relation to the other cardiac segments may contribute to a better understanding of the morphogenetic complexity of these phenotypes.
